# Leucyl-tRNA synthetase regulates casein synthesis in dairy cows via the mTORC1-LAT1 pathway

**DOI:** 10.5713/ab.24.0711

**Published:** 2025-02-27

**Authors:** Yongding Ke, Ximeng Du, Binglan Chen, Xi Chen, Chengchuang Song, Xingtang Fang, Yanhong Wang, Chunlei Zhang

**Affiliations:** 1 Institute of Cellular and Molecular Biology, School of Life Science, Jiangsu Normal University, Xuzhou, Jiangsu, China

**Keywords:** Large Neutral Amino Acid Transporter, Leucine, Leucyl-tRNA Synthetase, mTORC1

## Abstract

**Objective:**

Leucyl-tRNA synthetase (LARS) is an essential multifunctional enzyme in mammals, pivotal in maintaining cellular protein and amino acid balance. It facilitates tRNA aminoacylation, initiating intracellular protein synthesis, and serves as an intracellular leucine sensor. The sensor function enables LARS to activate the mTORC1 pathway via Rag GTPase binding, playing a critical role in the regulation of protein synthesis. Despite its significance, the precise mechanisms of these functions are yet to be fully delineated. This study examines LARS and its role in modulating milk protein synthesis.

**Methods:**

This study utilizes stable bovine mammary epithelial cell lines, LARS overexpression and LARS knockdown, validated by using Cell Counting Kit-8, Click-iT EdU, Western blot, real-time quantitative polymerase chain reaction, and immunoconfocal techniques.

**Results:**

Our findings show that LARS overexpression in bovine mammary epithelial cells (MAC-T) enhances cell proliferation and resultes intracellular leucine levels, thereby increasing casein production through the mTORC1 pathway. LARS enhances casein expression via the mechanistic Target of Rapamycin Complex 1, L-type Amino Transporters 1 (mTORC1-LAT1) pathway. This interaction is supported by a positive feedback mechanism from LAT1, enhancing the activation of the mTORC1 pathway. Additionally, LARS overexpression leads to increased LAT1 expression, improved LAT1 stability, and augmented its localization at the membrane. Our research indicates that LARS’s enhancement of LAT1 expression is contingent on its dual roles in translation and leucine sensing, whereas its impact on LAT1 localization is exclusively dependent on its leucine sensing function.

**Conclusion:**

LARS regulates LAT1 expression and membrane positioning through the mTORC1 pathway by detecting intracellular leucine levels, thereby influencing casein synthesis. These insights lay a theoretical groundwork for enhancing milk protein production and offer novel strategies for improving the quality of dairy products.

## INTRODUCTION

Milk is not only highly nutritious but also provides crucial immune protection to newborns before their own immune systems fully mature, ensuring their normal growth and development. It stands out as an ideal food due to its high nutritional content, ease of digestion and absorption, and convenience [1]. Milk protein and milk fat are vital sources of nutrition and energy for newborn animals and are pivotal in assessing emulsion quality [2]. Milk protein delivers essential amino acids necessary for bodily functions and plays roles in immune protection and microbial flora regulation. Casein, the primary milk protein comprising about 78% of the total protein content, binds phosphorus and calcium and is present at approximately 25 g/L in milk. The polymorphism of casein is a significant attribute that requires careful consideration in dairy production processes. Consequently, it is crucial to deepen our understanding of the lactation mechanisms in dairy cows and investigate the molecular processes regulating milk protein synthesis to enhance milk production and improve dairy product quality.

Leucine, a fundamental building block of proteins, primarily functions as a raw material in protein synthesis. Recent studies have indicated that the uptake of some amino acids by mammary epithelial cells exceeds the needs of milk protein synthesis [3]. Beyond serving as substrates for cellular growth and protein production, these amino acids also provide metabolic intermediates and energy [4–6], and act as active agents in the regulation of cell signaling factors, playing a crucial role in the synthesis and secretion of milk protein [7,8]. Leucyl-tRNA synthetase (LARS) is a pivotal enzyme in protein synthesis, traditionally known for its role in using adenosine triphosphate (ATP) to catalyze the attachment of leucine to its corresponding tRNA, thus forming leucyl-tRNA-an essential substrate for translation. Recently, the role of LARS as an intracellular leucine sensor has garnered significant research interest. In the presence of leucine, LARS can detect intracellular leucine levels, facilitating its translocation to lysosomes and promoting the conversion of Rag D-GTP to Rag D-GDP. This action enhances the localization of mTORC1 on lysosomes [9]. Leucine not only serves as a critical intracellular signaling molecule influencing milk protein synthesis and mammary cell proliferation [10,11], but also highlights the ongoing research into LARS’s ability to sense intracellular leucine levels and regulate protein synthesis, though the exact mechanisms remain to be elucidated.

Leucine, a polar molecule, typically has a higher intracellular than extracellular concentration, meaning it cannot freely cross the cell membrane and requires transporters for cell entry. In animals, leucine is primarily transported by the L-type amino acid transporter (LAT) family [12], with Large Neutral Amino Acid Transporter (LAT1) being the most crucial transmembrane protein. LAT1 expression is modulated by the mTORC1 pathway. It is known to accumulate on the lysosomal membrane, facilitating leucine’s entry into lysosomes. This process leads to the recruitment and activation of the mTORC1 complex on the lysosomal membrane via Rag GTPase, which triggers the phosphorylation pathway of mTORC1 [13,14]. LARS and LAT1 are key to maintaining protein and amino acid homeostasis within cells, regulating protein synthesis through distinct mechanisms. However, the specific mechanisms by which they regulate protein synthesis remain poorly understood, and their potential mutual regulatory effects have not yet been documented. This study aims to elucidate the molecular mechanism through which LARS regulates milk protein synthesis, further contributing to our understanding of these processes.

## MATERIAL AND METHODS

### Cell lines and storage conditions

The cells used in this experiment were bovine mammary epithelial cells (MAC-T) and human renal epithelial cell line HEK 293T cells. MAC-T cells were provided by Shandong Academy of Agricultural Sciences, and HEK 293T was purchased from American Type Culture Collection and stored in liquid nitrogen.

### Cell culture and transfection

Both MAC-T cells and HEK 293T cells were cultured in Dulbecco’s Modified Eagle Medium (DMEM) high glucose medium containing 10% fetal bovine serum, 1% penicilin-streptomycin solution and placed in a constant temperature incubator containing 5% CO_2_ at 37°C. When the MAC-T cell density reached 90%, the cells were sub-cultured and expanded. In this study, insulin-prolactin-hydrocortisone was used to induce milk protein secretion. When the cells grew to a stable state, the cells were changed to a differentiation medium (DMEM high glucose medium containing 10% fetal bovine serum, 1% penicillin-streptomycin solution, 5 μg/mL insulin, 1 μg/mL hydrocortisone and 2 ng/mL prolactin). The medium was changed daily, and cell status was observed during the procedures described above.

### Construction of stable cell lines

Both LARS-overexpression plasmid and LARS-knockdown plasmid were synthesized by Sai-ye Biotechnology Company (Guangzhou, China). When HEK 293T cell density reached 60%, plasmids were transfected into 293T cells using Polyethylenimine transfection reagent. Viral fluid was collected at two time points, 48 h and 72 h after transfection. When the MAC-T cell density reached 60%, polybrene was used to mediate virus infection of MAC-T cells. The expression of green fluorescent protein was observed between 48 h and 72 h after infection. When there was obvious green fluorescence in the cells, the infected cells were expanded and screened by puromycin.

### Cell counting kit-8 assay

In this experiment, cell viability was measured as an indicator of cell proliferation. The effect of LARS on the proliferation of MAC-T cells was detected by cell counting kit-8 (CCK-8). After MAC-T cells grew to a stable state, they were sub-cultured in 96-well plates and continued to grow for 24 h, an average of 3–5×10^4^ cells were inoculated per well. 10 μL of CCK-8 solution was added to 100 μL of culture medium and incubated in the dark for 1 h. Then the absorbance was measured at 450 nm using a Infinite F50 Plus microplate reader from TECAN (Männedorf, Swiss; n = 6).

### 5-Ethynyl 2′-deoxyuridine assay

In this experiment, cell proliferation was detected by observing the number of 5-ethynyl 2′-deoxyuridine (EdU) staining positive cells. After MAC-T cells had grown to a steady state, they were sub-cultured in 24-well plates and continued to fluorescence microscope from OLYMPUS U-RFL-T grow for 24 h. The proliferation medium was changed to complete medium containing EdU reagent (the ratio of EdU to proliferation medium was 1:1,000), and the transfected cells were incubated for 2 h in the dark 24 h after transfection. The cells were then fixed and stained, and the cell growth was observed and recorded using a fluorescence microscope.

### Quantitative real-time polymerase chain reaction

Total cellular RNA was extracted from cultured MAC-T using Trizol according to the manufacturer’s instructions, followed by reverse transcription of RNA into cDNA using the Akora Bio Evo M-MLV reverse transcription kit (Eric Biotechnology, Hunan, China). Relative mRNA expression was then measured by quantitative reverse transcription polymerase chain reaction (qRT-PCR) on an ABI Step One Plus real-time PCR instrument using the SYBR Green Pro Taq HS Premix qPCR kit (Eric Biotechnology). In this study, the NCBI website and Primer 3 online design website were used to design qRT-PCR primers for LARS and other genes, and GAPDH was used as an internal control. The relative expression levels of genes were detected by 2^−ΔΔCt^ method. The relevant primers used in this study were synthesized by ShangHai Sangon Biotech (Shanghai, China), as shown in Table 1.

### Western blot assay

Cellular proteins were lysed using RIPA protein extraction reagent. The concentration of protein samples was determined using the bicinchoninic acid Protein Concentration Assay kit. The processed protein samples were separated by 10% sodium dodecyl sulfate polyacrylamide gel electrophoresis, transferred to polyvinylidene fluoride membranes, blocked with 5% skim milk, and incubated with primary antibodies against β-actin (66009-1-Ig, 1:50,000; Proteintech, Wuhan, China), LARS (BS72681, 1:1,000; Bioworld), LAT1 (5347S, 1:1,000; Cell Signaling Technology, Danvers, MA, USA), 4F2hc (15193-1-AP, 1:10,000; Proteintech), ATP1A1 (14418-1-AP, 1:10,000; Proteintech), mTOR (66888-1-Ig, 1:25,000; Proteintech), S6K (14485-1-AP, 1:4,000; Proteintech), p-S6K (28735-1-AP, 1:4000; Proteintech), α-casein (ER1905-45, 1:1000; HUABIO, Woburn, MA. USA), β-casein (bs-23551R, 1:1000; Bioss, Woburn, MA, USA). After washing three times with 1×tris-buffered saline with Tween 20, polyvinylidene fluoride membranes were incubated with secondary antibodies conjugated to horseradish peroxidase. After incubation with enhanced chemiluminescence reagent, the imaging exposure was performed using a protein gel imager from e-BLOT Touch imager. Image J software was used to analyze the gray value of the protein bands.

### Immunofluorescence assay

MAC-T cells grown to a steady state were sub-cultured on cell slides in 24-well plates, washed twice with phosphate-buffered saline (PBS), and fixed with 100% cold methanol for 10 min at 4°C. After washing three times with PBS, the solution was blocked with 5% bovine serum albumin and incubated for 30 min at room temperature on a shaker. After washing three times with PBS, cells were incubated with the primary antibody (LARS 1:100; Bioworld, Nanjing, China) overnight at 4°C. After incubation with the primary antibody, the cells were washed with PBS and incubated with coralite594-conjugated secondary antibody (1:100; Proteintech) for 2 h. Nuclei were stained with 4′,6-diamidino-2-phenylindole (DAPI) solution. After washing three times with PBS, cell slides were inverted onto slides dripped with anti-fluorescence quench and viewed by laser confocal microscopy from Yohogawa CV1000.

### Leucine starvation and stimulation of cells

For leucine starvation, cells were washed twice with PBS, then incubated with specially tailored high glucose DMEM medium without leucine for 4 h to deplete leucine, and then stimulated with leucine solution again for 2 h.

### Statistical analysis

All data were compared between the two groups using T test. Statistical analysis and mapping were performed using GraphPad Prism5 software, and all data are presented as the mean±standard error of the mean (SEM) of at least three independent experiments. *represents p<0.05; **represents p<0.01; ***represents p<0.001.

## RESULTS

### LARS promotes the proliferation of MAC-T cells

**Figure 1:**
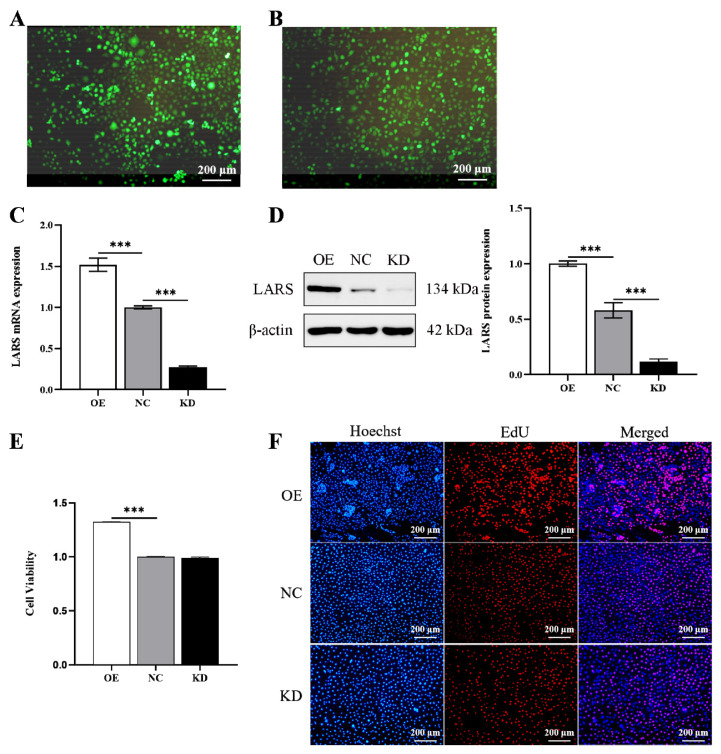
LARS promotes the proliferation of MAC-T cells. (A) Expression of green fluorescent protein in LARS-OE stable cell lines. (B) Expression of green fluorescent protein in LARS-KD stable cell lines. (C) The mRNA expression of *LARS* were examined by qRT-PCR. (D) Western blot analysis detected the protein levels of LARS and statistical analysis of the gray values of protein bands. (E) Cells proliferation of MAC-T cells were detected by CCK-8. (F) Cells proliferation of MAC-T cells were detected by EdU. Data are representative of the means±SEM of three independent experiments. Values are the mean±SEM for three biological replicates, *** p<0.001. LARS, leucyl-tRNA synthetase; OE, overexpression; NC, negative control; KD, knockdown; qRT-PCR, quantitative reverse transcription polymerase chain reaction; CCK-8, cell counting kit-8; SEM, standard error of the mean.

To investigate the regulatory function of LARS in MAC-T cells, LARS overexpressing (LARS-OE) and LARS knockdown (LARS-KD) stable cell lines were constructed using lentivirus packaging technology (Figures 1A–1D). Since the number and activity of mammary epithelial cells are crucial determinants of milk production, we also assessed the impact of LARS manipulation on MAC-T cell proliferation. The results showed that the cell proliferation was significantly increased in the LARS-OE cells, though the cell viability of the LARS-KD cell lines did not change significantly (Figures 1E, 1F). These results suggest that LARS plays a pivotal role in promoting the proliferation of MAC-T cells.

### LARS regulates casein synthesis via mTORC1

**Figure 2:**
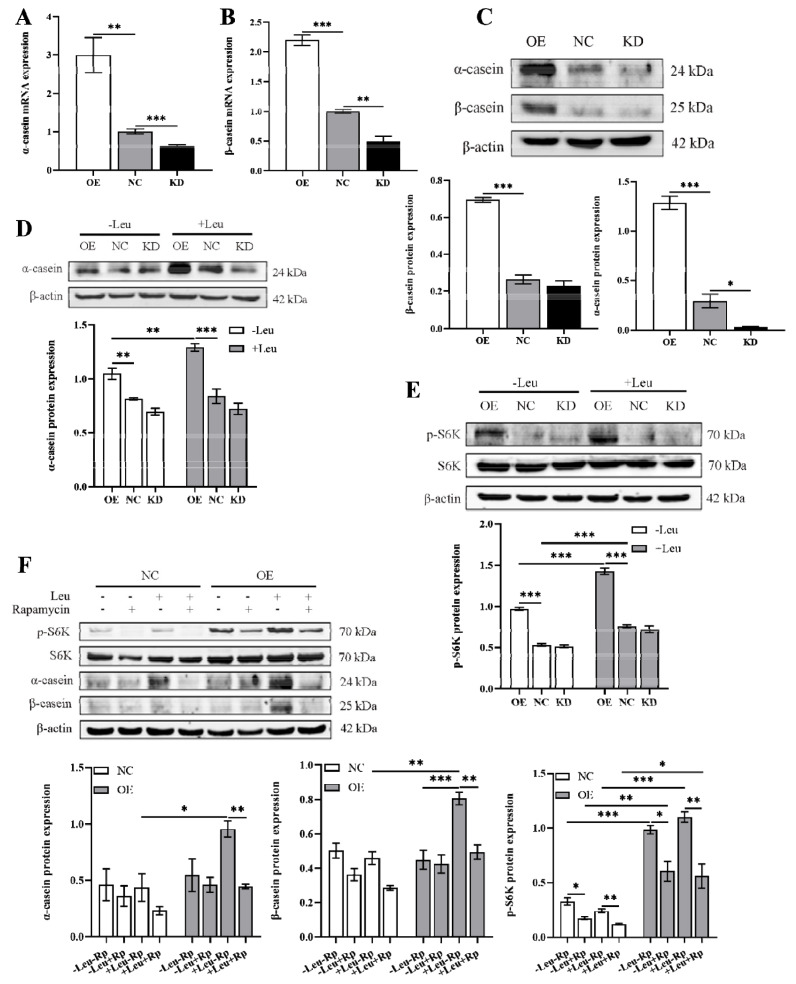
LARS regulate casein synthesis via mTORC1. The mRNA expression of *α-casein* (A) and *β-casein* (B) were examined by qRT-PCR at 48 h. (C) Western blot analysis detected the protein levels of α-casein and β-casein at 48 h, and statistical analysis of the gray values of protein bands. (D) Western blot analysis detected the protein levels of α-casein at 48 h of leucine-starved culture and leucine-sufficient culture, and statistical analysis of the gray values of protein bands. (E) Western blot analysis detected the phosphorylation of S6K at 24 h of leucine-starved culture and leucine-sufficient culture, and statistical analysis of the gray values of protein bands. (F) Western blot analysis detected the protein levels of α-casein and β-casein and the phosphorylation of S6K after treatment with rapamycin and leucine for 24 h, and statistical analysis of the gray values of protein bands. +Leu means that the cells were deprived of leucine followed by its readdition to the medium. Data are representative of the means±SEM of three independent experiments. Values are the mean±SEM for three biological replicates, * p<0.05, ** p<0.01, *** p<0.001. OE, overexpression; NC, negative control; KD, knockdown; LARS, leucyl-tRNA synthetase; qRT-PCR, quantitative reverse transcription polymerase chain reaction; SEM, standard error of the mean.

We discovered that LARS stimulates the proliferation of bovine mammary epithelial cells and sought to determine its influence on milk protein synthesis. To do this, we utilized an *in vitro* lactation model. Our results demonstrated a notable increase in both mRNA and protein levels of α-casein and β-casein in the LARS-OE cells (Figures 2A–2C). Conversely, in the LARS-KD cell lines, the mRNA levels of both caseins were significantly reduced (Figures 2A, 2B). While the protein expression of α-casein decreased, β-casein levels remained unchanged (Figure 2C). These findings suggest that overexpression of LARS enhances both the transcription and translation of α-casein in bovine mammary epithelial cells, thereby potentially boosting milk protein synthesis.

Given LARS’s role as an intracellular leucine sensor, we proceeded to assess its impact on milk protein synthesis under conditions of either leucine is adequate or deficient. Our experiments demonstrated that leucine addition significantly boosted casein expression in both wild-type and LARS-OE cells, with LARS-OE cells showing an even greater enhancement of casein expression in response to leucine (Figure 2D). These findings indicate that LARS mediates the effects of intracellular leucine levels to regulate milk protein synthesis effectively.

Previous studies have indicated that both leucine and LARS can influence the activation of the mTORC1 pathway. Building on this, we investigated LARS’s role in mTORC1 pathway regulation. Our results revealed that while leucine had no significant effect on total S6K protein expression across three cell lines, it significantly enhanced the phosphorylation of S6K. Moreover, LARS-OE further augmented S6K phosphorylation in response to leucine. Conversely, leucine did not significantly affect S6K phosphorylation when LARS was knocked down (Figure 2E). This suggests that LARS is crucial in mediating intracellular leucine levels to regulate mTORC1 activation. To probe whether LARS influences milk protein synthesis via the mTORC1 pathway, we employed rapamycin to inhibit mTORC1 activity. Upon adding rapamycin, there was a significant reduction in both casein expression and S6K phosphorylation in wild-type and LARS-OE cells (Figure 2F). Despite this inhibition, an increase in casein expression persisted in LARS-OE cells, hinting at additional pathways through which LARS may regulate casein synthesis. Overall, our results suggest that LARS can regulate casein synthesis through mTOR pathway, but it does not exclude that LARS regulates casein synthesis through other pathways.

### LARS promoted casein synthesis via mTORC1-LAT1

**Figure 3:**
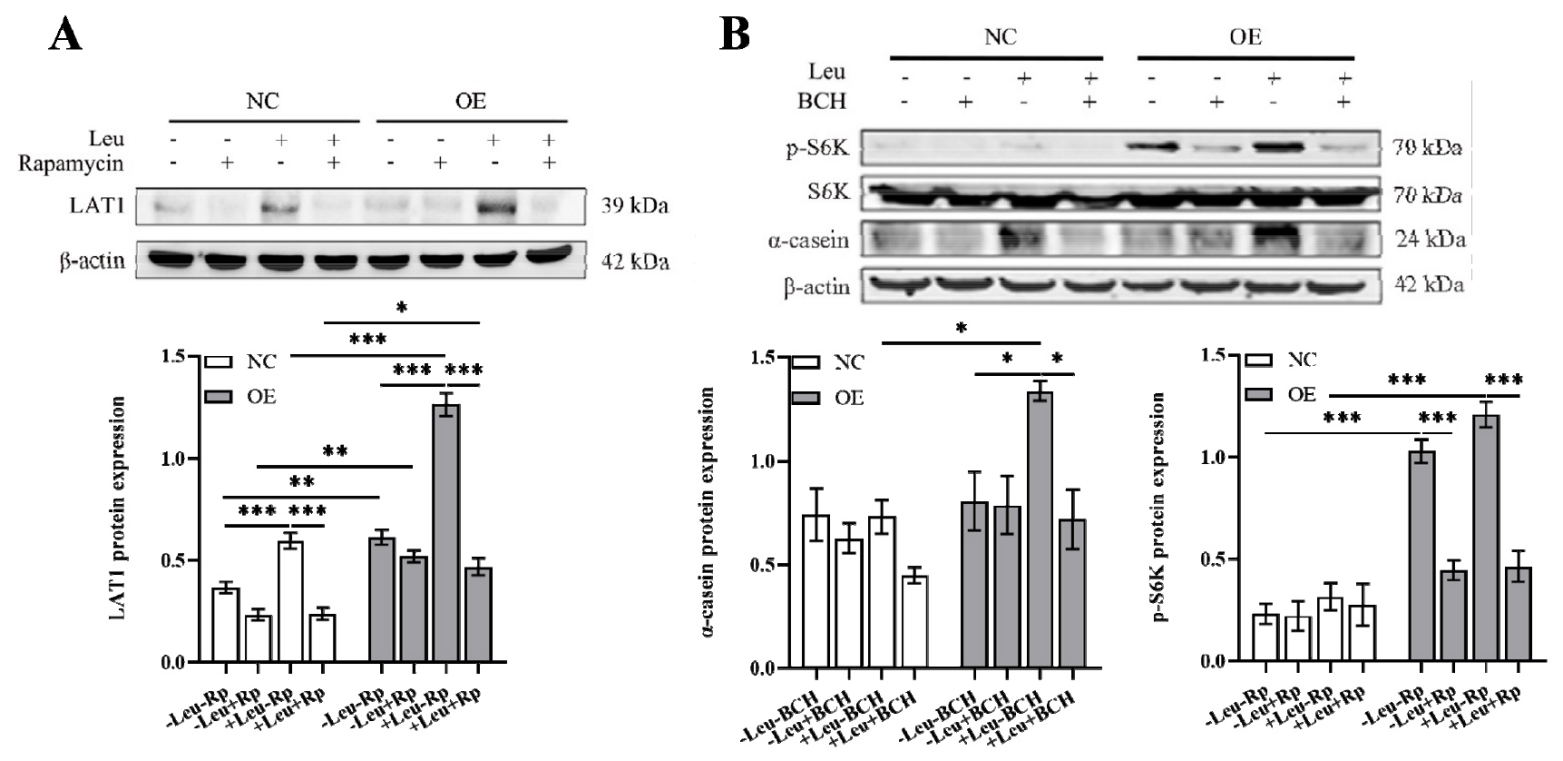
LARS promoted casein synthesis via mTORC1-LAT1. (A) Western blot analysis detected the protein levels of LAT1 after treatment with rapamycin and leucine for 24 h, and statistical analysis of the gray values of protein bands. (B) Western blot analysis detected the casein expression levels and phosphorylation of S6K after treatment with BCH and leucine for 24 h, and statistical analysis of the gray values of protein bands. Data are representative of the means±SEM of three independent experiments. Values are the mean±SEM for three biological replicates, * p<0.05, ** p<0.01, *** p<0.001. NC, negative control; OE, overexpression; LAT1, large neutral amino acid transporter; LARS, leucyl-tRNA synthetase; SEM, standard error of the mean.

Previous research has established that LARS can convey leucine signals to the mTORC1 pathway, thus enhancing the activation of mTORC1 signaling which promotes casein synthesis. However, leucine, after being ingested by animals, is distributed in the extracellular fluid and cannot freely traverse cell membranes. Instead, it requires active transport into cells via the LAT family, facilitated by ATP against the concentration gradient. To further explore how the mTORC1 pathway regulates casein synthesis, rapamycin was employed to inhibit mTORC1 activation, and the expression of LAT1 was monitored. Our experiments showed that in wild-type cells, leucine promoted LAT1 expression, which was almost completely inhibited by rapamycin (Figure 3A). In LARS-OE cells, LAT1 expression was significantly elevated compared to that in wild-type cells, and further increased with the addition of leucine. When rapamycin was added, although LAT1 expression was significantly reduced, it remained higher in LARS-OE cells compared to wild-type cells. Interestingly, leucine addition did not alter LAT1 expression in either wild-type or LARS-OE cells post-rapamycin treatment (Figure 3A). These findings suggest that rapamycin disrupts the regulation of LAT1 expression by leucine, demonstrating that LARS-mediated intracellular leucine levels regulate LAT1 expression through the mTORC1 pathway.

To investigate whether LARS-mediated regulation of casein synthesis is dependent on LAT1 activity, we utilized BCH, a selective inhibitor of LAT1, to inhibit its activity. Our results indicate that LARS-OE significantly boosted α-casein expression in the presence of leucine (Figure 3B). However, this upregulation of casein by both leucine and LARS was significantly curtailed with the addition of BCH (Figure 3B), suggesting that LARS’s effect on casein synthesis is contingent on LAT1 activity. Further analysis was conducted on the impact of LAT1 on the phosphorylation level of S6K. We observed that in wild-type cells, S6K phosphorylation was minimal and was nearly completely abolished following BCH treatment (Figure 3B). In LARS-OE cells, however, S6K phosphorylation levels were notably higher, and leucine further enhanced this phosphorylation. Yet, these levels were significantly reduced upon BCH administration (Figure 3B), indicating that LAT1 inhibition markedly affects the activation of the mTORC1 pathway. These findings underscore that LAT1 not only regulates mTORC1 pathway activity but is also essential for LARS’s role in mediating intracellular leucine levels to regulate LAT1 expression through the mTORC1 pathway. This interaction, in turn, positively influences the activation of the mTORC1 pathway, ultimately promoting casein synthesis.

### LARS functions as a leucine sensor to promote LAT1 expression and membrane localization

**Figure 4:**
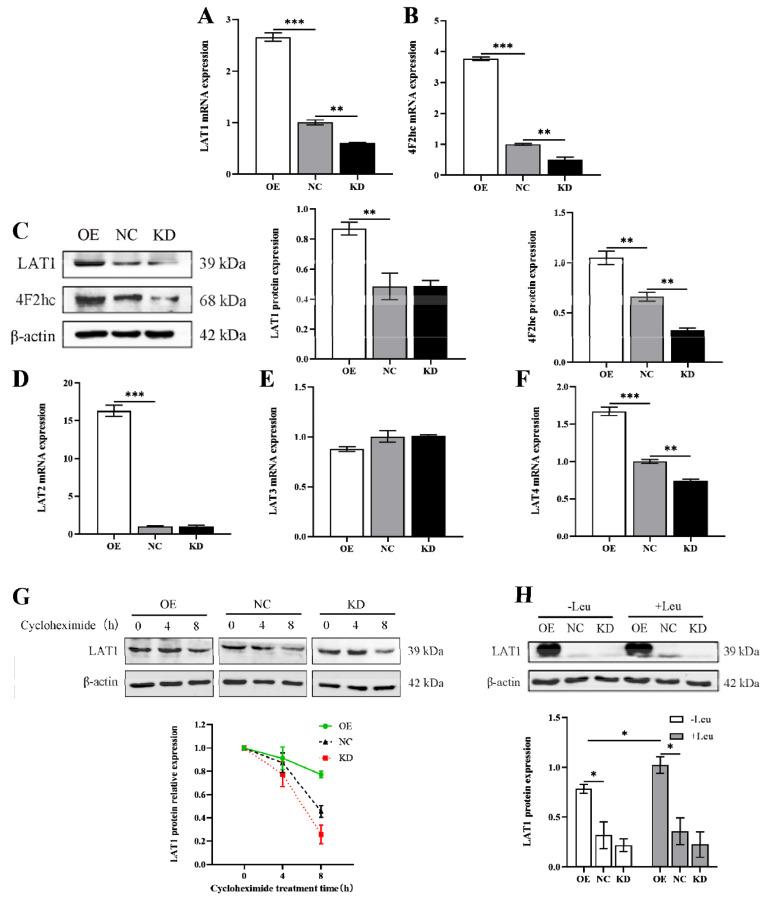
LARS functions as a leucine receptor to promote LAT1 expression and membrane localization. The mRNA expression of *LAT1* (A) and *4f2hc* (B) were examined by qRT-PCR. (C) Western blot analysis detected the protein levels of LAT1 and 4f2hc, and statistical analysis of the gray values of protein bands. The mRNA expression of *LAT2* (D), *LAT3* (E) and *LAT4* (F) were examined by qRT-PCR. (G) Western blot analysis detected the protein levels of LAT1 after cycloheximide treatment for 0 to 8 h, and statistical analysis of the gray values of protein bands. (H) Western blot analysis detected the protein levels of LAT1 at 24 h of leucine-starved culture and leucine-sufficient culture, and statistical analysis of the gray values of protein bands. Data are representative of the means±SEM of three independent experiments. Values are the mean±SEM for three biological replicates, * p<0.05, ** p<0.01, *** p<0.001. LAT1, large neutral amino acid transporter; OE, overexpression; NC, negative control; KD, knockdown; LARS, leucyl-tRNA synthetase; qRT-PCR, quantitative reverse transcription polymerase chain reaction.

Previous studies have demonstrated that LARS regulates casein synthesis via LAT1. Our current experiments aimed to elucidate the specific mechanisms underlying LARS’s influence on LAT1. We found that LARS significantly enhanced the transcription and translation of LAT1 (Figures 4A, 4C). The protein 4F2hc, which is crucial for forming heterodimers with LAT1 and enhancing its membrane localization and stability [15,16], showed a pattern of expression that mirrored LAT1 (Figures 4B, 4C). Additionally, we analyzed the gene expression levels of other proteins in the LAT family. The results indicated that LARS-OE notably increased the mRNA levels of LAT2 and LAT4, while LARS knockdown significantly reduced LAT4 gene expression (Figures 4D, 4F). LAT3 expression, however, remained unaffected by changes in LARS (Figure 4E). Given the correlation between LARS activity and 4F2hc expression, we hypothesized that LARS could also enhance LAT1 stability. To test this, we treated cells with cycloheximide to block new protein synthesis and observed the degradation rates of LAT1. The degradation was significantly slower in LARS-OE cells, whereas it accelerated in LARS-KD cells (Figure 4G). The line chart clearly illustrated that LAT1 degradation was slowest in LARS-OE cells (Figure 4G). These findings suggest that LARS not only boosts LAT1’s transcription and translation but also enhances its protein stability, thereby increasing LAT1’s overall expression level within the cells. This multifaceted regulatory approach underscores LARS’s pivotal role in managing cellular amino acid transport and protein synthesis.

Next, we incorporated leucine as a variable to further explore whether its regulation on LAT1 is mediated through LARS. The results indicated that adding leucine further enhanced the upregulation of LAT1 protein expression driven by LARS (Figure 4H). This suggests that LARS can indeed mediate intracellular leucine levels to promote LAT1 expression, affirming its role in the regulatory pathway.

**Figure 5:**
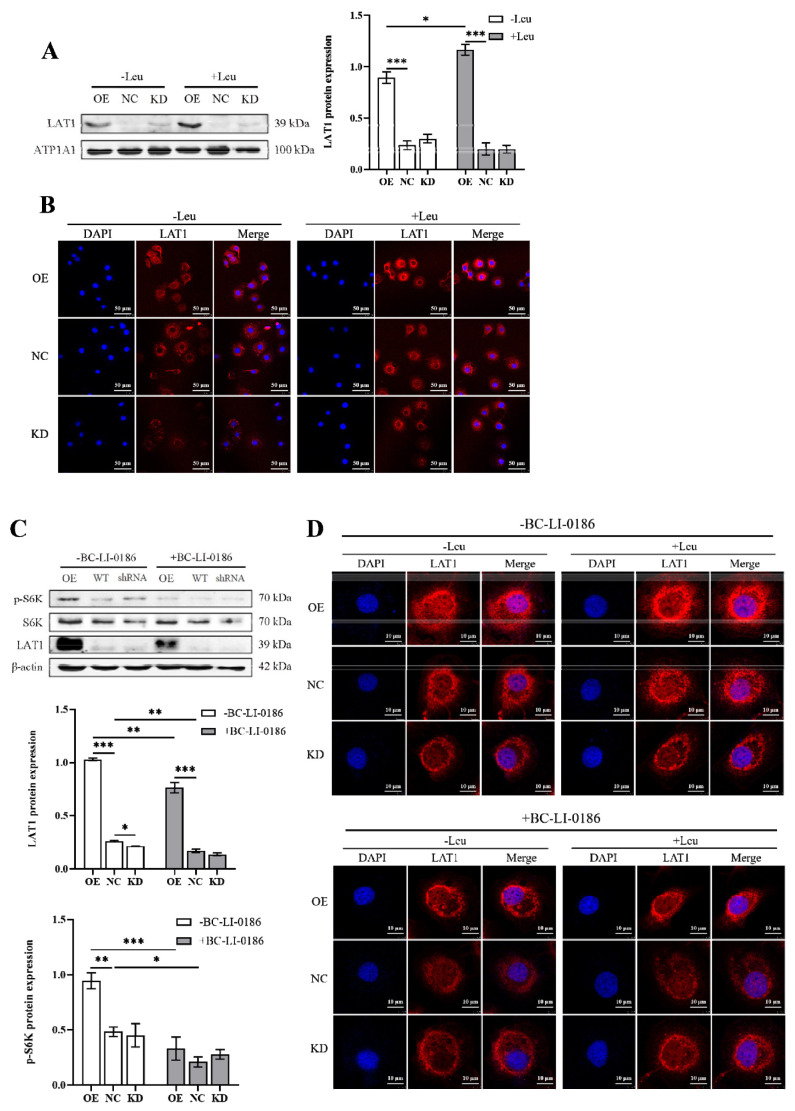
LARS functions as a leucine receptor to promote LAT1 expression and membrane localization. (A) MAC-T cells were starved of leucine for 4 h and stimulated with the addition of 0.9 mM leucine for 1h, then western blot analysis detected the protein levels of LAT1 in the cell membrane, and statistical analysis of the gray values of protein bands. (B) MAC-T cells were starved of leucine for 4 h and stimulated with the addition of 0.9 mM leucine for 1h, then immunofluorescence assay was used to detect the subcellular distribution of LAT1. (C) Western blot analysis detected the protein levels of LAT1 and the phosphorylation of S6K after treatment with BC-LI-0186 for 24 h, and statistical analysis of the gray values of protein bands. (D) MAC-T cells were starved of leucine for 4 h with BC-LI-0186 and stimulated with the addition of 0.9 mM leucine for 1h with BC-LI-0186, then immunofluorescence assay was used to detect the subcellular distribution of LAT1. Data are representative of the means±SEM of three independent experiments. Values are the mean±SEM for three biological replicates, * p<0.05, ** p<0.01, *** p<0.001. OE, overexpression; NC, negative control; KD, knockdown; LAT1, large neutral amino acid transporter; DAPI, 4′,6-diamidino-2-phenylindole; LARS, leucyl-tRNA synthetase; SEM, standard error of the mean.

LAT1, a key intracellular leucine transporter, primarily functions at the cell membrane to facilitate the uptake of extracellular leucine. We assessed the expression level of LAT1 on the cell membrane and found that it was significantly higher in LARS-OE cells compared to wild-type cells. Additionally, the presence of leucine further augmented LAT1 expression on the membrane (Figure 5A), suggesting that LARS may enhance the translocation of LAT1 to the cell membrane. To further explore LAT1’s intracellular distribution, we performed a cellular immunofluorescence confocal assay. Under conditions of leucine starvation, there was no significant change in LAT1 membrane expression between wild-type and LARS-OE cells. However, upon leucine stimulation, LAT1’s localization to the membrane increased in both cell lines, with a more pronounced effect observed in LARS-OE cells (Figure 5B). In contrast, LAT1 expression at the membrane was markedly low in LARS-KD cells, regardless of leucine presence. These findings underscore that LARS not only promotes LAT1 translocation to the membrane but also mediates the intracellular leucine levels to regulate this process, enhancing LAT1 membrane localization (Figure 5B).

Our previous studies established that LARS mediates intracellular leucine levels and influences both the expression and localization of LAT1. LARS possesses both classical translational functions and non-translational roles as a leucine sensor. In our earlier studies using LARS knockdown cell lines, where total LARS protein expression was significantly reduced, it was challenging to ascertain which specific function of LARS was involved. To dissect these roles, we used BC-LI-0186 to specifically inhibit the leucine sensor function of LARS and investigate its mechanisms in regulating LAT1 and the mTORC1 pathways. The results indicated that treatment with BC-LI-0186 almost completely inhibited LAT1 expression and S6K phosphorylation in wild-type cell lines (Figure 5C). In LARS-OE cells, BC-LI-0186 treatment markedly reduced S6K phosphorylation and significantly but not completely inhibited LAT1 protein expression, which remained notably higher than in wild-type cells (Figure 5C). These findings suggest that LARS’s activation of the S6K pathway is entirely reliant on its function as a leucine sensor, while the regulation of LAT1 expression depends on both the classical translational functions and leucine sensing capabilities of LARS. Further analysis of LAT1 subcellular distribution post BC-LI-0186 treatment showed a significant decrease in LAT1 membrane localization in both wild-type and LARS-OE cells, regardless of leucine presence (Figure 5D). Additionally, the subcellular distribution of LAT1 did not exhibit notable changes upon leucine addition in either cell line (Figure 5D). In LARS-KD cell lines, LAT1 membrane localization remained unchanged regardless of BC-LI-0186 treatment (Figure 5D). These results indicate that the regulation of LAT1’s subcellular distribution by LARS is almost entirely dependent on its leucine sensor function, as BC-LI-0186 substantially blocks the effects of both leucine and LARS on LAT1 localization.

## DISCUSSION

Studies have highlighted the pivotal role of LARS in regulating the mTORC1 pathway, a crucial cellular hub for managing nutrition, cell proliferation, protein synthesis, and other vital cellular activities. It has been observed that LARS is essential for the viability and proliferation of TSC-null cells, with LARS inhibitors noted to decrease cell proliferation and survival [17]. Similarly, a study involving bovine mammary epithelial cells demonstrated that LARS knockdown led to reduced cell proliferation and a decreased frequency of cells in the S and G2/M phases, aligning with our findings where LARS-OE notably increased cell proliferation. However, intriguingly, LARS knockdown did not significantly impact cell proliferation. This observation suggests that long-term stability of the knockdown in established cell lines might trigger compensatory intracellular mechanisms that mitigate the effects of LARS reduction on proliferation. Thus, while LARS knockdown showed no substantial effect on cell proliferation, it is clear from the broader body of evidence that LARS generally promotes the proliferative activity of bovine mammary epithelial cells. This complexity indicates the presence of other regulatory layers that may compensate for the loss of LARS function, highlighting the nuanced role of LARS in cellular growth regulation.

It has been established that LARS can regulate the synthesis of proteins in the cytoplasm, either directly or indirectly. Previous findings indicated that knocking out LARS inhibits the expression of β-casein in dairy cow mammary epithelial cells. Our study corroborated these findings, showing that LARS enhances casein synthesis in bovine mammary epithelial cells. Interestingly, the mRNA level of β-casein in the LARS knockdown group was significantly decreased, but the protein level was not down-regulated. The cause of this phenomenon, on the one hand, the time of the eukaryotic gene expression of transcription and translation and site is the interval of time and space, on the other hand, after transcription, there will be post-transcriptional processing, degradation of transcription products, translation, post-translational processing and modification of several levels [18]. The process of protein synthesis usually takes a certain amount of time, while mRNA expression can respond more quickly to external stimuli. Therefore, in some cases, changes in mRNA may appear earlier than changes in protein levels, leading to an inconsistent trend between the two [19]. Leucine, a crucial branched-chain essential amino acid in poultry and mammals, significantly impacts skeletal muscle formation [20], inhibits protein degradation in the mammary gland, and boosts mammary protein synthesis [21]. Notably, leucine activates the mTORC1 signaling pathway more effectively than other amino acids [22]. However, amino acid signaling does not act directly on the mTORC1 pathway but influences it indirectly through various protein interactions [23]. LARS plays a vital role as an intracellular leucine sensor, detecting changes in intracellular leucine levels. In a leucine-rich environment, LARS catalyzes the conversion of GTP to GDP in the RagD subunit of Rag GTPase, which activates the RagB/RagD heterodimer, promoting the translocation of mTORC1 to lysosomal surfaces and activating the pathway. In this study, we combined variables of leucine and LARS to further investigate their specific mechanisms on milk protein synthesis and the mTORC1 pathway. Consistent with earlier findings, our research confirmed that both leucine and LARS enhance casein synthesis and the phosphorylation of the mTORC1 pathway in bovine mammary epithelial cells. Overexpression of LARS further enhanced the stimulatory effect of leucine on milk protein synthesis and mTORC1 pathway activation. Using rapamycin to inhibit the mTORC1 pathway, we observed that LARS and leucine’s regulation of casein synthesis was notably reduced. However, even with rapamycin treatment, leucine continued to promote casein synthesis compared to wild-type cells, suggesting additional mechanisms by which LARS regulates casein synthesis. Given LARS’s classical role in promoting the covalent linkage of leucine to its corresponding tRNA, it is plausible that LARS-OE could directly initiate translation by enhancing tRNA aminoacylation, thus promoting casein synthesis alongside its effects through the mTORC1 pathway. In conclusion, LARS mediates intracellular leucine levels to regulate milk protein synthesis primarily through the mTORC1 pathway.

The transport of amino acids into cells is facilitated by specific transporters, among which LAT1 plays a crucial role. LAT1 catalyzes the transmembrane transport of large neutral amino acids in a manner that is independent of sodium ions and pH, providing essential amino acids to cells. While much of the previous research on LAT1 has been centered on its role in cancer pathology, its involvement in protein synthesis, particularly in the interaction with LARS, has not been extensively reported. Previous studies have demonstrated that LAT1 is highly expressed in the mammary glands of lactating rats and pigs and is closely associated with milk protein production, underscoring its importance in amino acid absorption through the mammary gland [24,25]. Moreover, mTORC1 signaling, which is critical for milk protein synthesis, is activated by inducing LAT1 expression in dairy cow mammary glands [26]. In our research, we discovered that LARS regulates casein synthesis via the mTORC1-LAT1 pathway, which in turn can positively feedback to activate the mTORC1 pathway. This study also reveals, for the first time, that LARS enhances LAT1 transcription and translation and inhibits its degradation, thus boosting overall LAT1 expression in cells. LAT1 forms a major part of the LAT1-4F2hc heterodimeric complex, functioning predominantly at the cell membrane. LAT1 localization on the plasma membrane of acinar epithelial cells has been observed, providing essential amino acids for the mammary gland. Additionally, it has been shown that prolactin can elevate LAT1 expression and activity by promoting its transfer from the cytosol to the plasma membrane in dairy cow mammary epithelial cells [27]. We also found that LARS promotes LAT1 translocation to the cell membrane in the presence of leucine. The use of BC-LI-0186, a functional inhibitor that disrupts the interaction between LARS and RagD GTPase, allowed us to ascertain that the regulation of LAT1 expression by LARS is contingent upon both its translational function and its role as a leucine sensor [28]. Furthermore, the subcellular localization of LAT1 regulated by LARS depends entirely on LARS’s ability to sense leucine. This nuanced understanding of LARS and LAT1 interaction opens potential pathways for enhancing milk protein synthesis through targeted cellular mechanisms.

This study delineates a comprehensive feedback loop involving leucine, LARS, mTORC1, and LAT1, crucial for regulating cellular processes. When extracellular leucine levels rise, LAT1 transports leucine into the cell, increasing intracellular leucine concentrations. LARS, sensing this increase, promotes the attachment of leucine to its cognate tRNA, initiating protein synthesis. Concurrently, LARS also facilitates the binding of leucine to Rag GTPase, enhancing the activation of the mTORC1 pathway. Activation of the mTORC1 pathway plays a dual role: it not only boosts intracellular protein synthesis and cell proliferation but also promotes the translocation of LAT1 to the cell membrane. This increase in LAT1 activity at the membrane enhances leucine transport into the cell, establishing a positive feedback loop that further stimulates the mTORC1 pathway. This mechanism ensures the maintenance of vital cellular functions and amino acid homeostasis, demonstrating a finely tuned regulatory system that responds dynamically to changes in cellular environment.

## CONCLUSION

**Figure 6:**
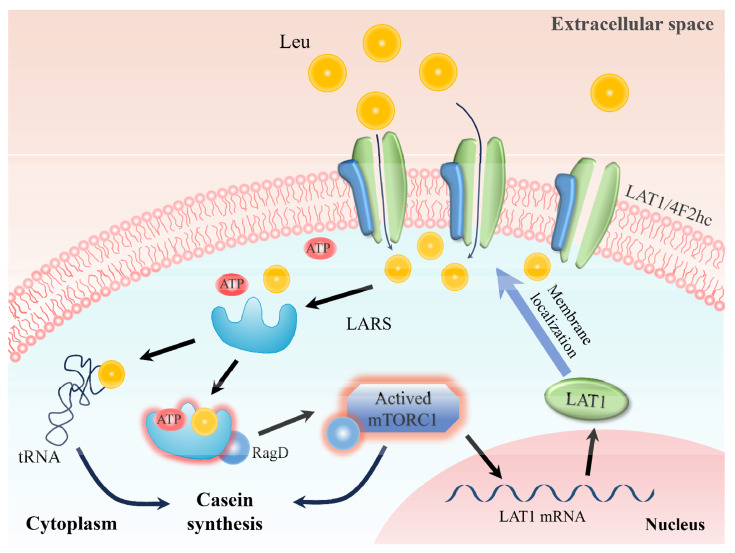
LARS regulates casein biosynthesis pattern through LAT1 in dairy cow mammary epithelial cells. After entering cells, leucine can be sensed and bound by LARS. On the one hand, it can promote casein synthesis by binding leucine to tRNA. On the other hand, it promotes casein synthesis by activating mTOR signaling pathway, and further promotes LAT1 transfer to the cell membrane. LARS, leucyl-tRNA synthetase; LAT1, large neutral amino acid transporter.

In summary, by detecting intracellular leucine levels, LARS regulates LAT1 expression and membrane positioning through the mTORC1 pathway, ultimately influencing casein synthesis. These insights lay a theoretical groundwork for advancing milk protein production and offer novel strategies for improving dairy product quality (Figure 6).

## Figures and Tables

**Table 1:** qRT-PCR primers

Primer name	Sequence (5′ to 3′)
*GAPDH*-F	GGGTCATCATCTCTGCACCT
*GAPDH*-R	GGTCATAAGTCCCTCCACGA
*LARS*-F	TTGACGATCCAGTGTTGGGG
*LARS*-R	ATCTGCCCGTAGCCCATTTT
*CSN1S1*-F	ACATCCTATCAAGCACCAAGGACTC
*CSN1S1*-R	GACGAAATGCTTTCAGCTTCCA
*CSN2*-F	AACAGCCTCCCACAAAAC
*CSN2*-R	AGCCATAGCCTCCTTCAC
*4F2hc*-F	ACCTAAAGGAGCGGATGGAT
*4F2hc*-R	AGGGTTCTGGCCCTTGTAGT
*LAT1*-F	ACCCTCACTGGTGTTCACG
*LAT1*-R	CTCCGGTTTCTGGTAGCG
*LAT2*-F	TCACCGGCGACATATACACG
*LAT2*-R	AGGTTGACCTTAATGGGGCG
*LAT3*-F	TGGCATCTGCCTAACCTTCT
*LAT3*-R	TAATGGCCATGAAGGACACA
*LAT4*-F	ACCCAAACTCCCTGTCTGTG
*LAT4*-R	TTGACTCCTGGGAAGGTGAC

qRT-PCR, quantitative reverse transcription polymerase chain reaction.
